# The Harms of Family-Oriented Informed Consent in Clinical Practice in Two Megacities in Northern China: a Sociological and Ethical Study

**DOI:** 10.1007/s41649-025-00373-1

**Published:** 2025-11-15

**Authors:** Jing-Ru Li, Simon Walker, Jing-Bao Nie

**Affiliations:** Bioethics Centre, University of Otago, Dunedin, New Zealand

**Keywords:** Medical practice in China, Family-oriented informed consent, Psychological resilience, Bad news, Truth-telling, Harm

## Abstract

In clinical practice in China, a family-oriented approach to informed consent is widely used. Under this approach, families may control whether patients are provided with information and make medical decisions for the patient without asking what they would want. One of the main justifications is its purported benefits for patients. Recent studies have begun to expose ways in which this approach may be more harmful than beneficial to patients. Integrating empirical investigation with normative analysis, this study examines the experienced and perceived benefits and harms of family-oriented informed consent by Chinese patients, family caregivers, and healthcare professionals from two megacities. Semi-structured and in-depth interviews were undertaken with 13 patients, 14 family caregivers, and 9 medical professionals in Tianjin and Beijing, two megacities in northern China. The findings of these interviews were thematically analysed. When taking the perspective of a caregiver, most of the participants across the three groups described the family-oriented approach as more beneficial to patients. However, when thinking of themselves as the patient, most participants believed that it would be better for them if they were provided with information and given an opportunity to decide. The interviews revealed some significant harms of family-orientated informed consent, such as delay in treatment, psychological abandonment, and suppression of self-management. This study challenges the widespread assumptions about the benefits of family-oriented informed consent. Some of the harms of this approach are unveiled and discussed. Two opposing attitudes toward this approach to informed consent, derived from the contrasting views of family members and patients, uncover the potential flaws in the beneficence-based justification for this practice.

## Introduction

Informed consent is a standard and routine part of clinical practice in most countries today. However, this was not the case until the latter part of the twentieth century (Bazzano et al. 2021). In a 1961 study, for example, 90% of American physicians did not routinely inform patients of cancer diagnosis, and one predominant reason for this practice was concern about patients “losing hope” (Oken 1961). While the norm of informed consent for biomedical research was established earlier, the doctrine of informed consent in clinical settings was not widely practiced until the 1970 s or later (Surbone et al. 2004; Oluchi 2013; Faden and Beauchamp 1986, 92). In America in the early 1970 s, a series of legal cases provoked fierce debates about a patient’s right to informed consent. These debates extended into the bioethical domain, and guided the writing of important position statements such as the American Medical Association (AMA) Code of Medical Ethics and the President’s Commission (Faden and Beauchamp 1986, 96). Since then, the importance of informed consent has been increasingly recognised and integrated into clinical practice.

In most Western counties, informed consent is focused on the rights of the individual. Competent patients have a legal right to decide whether and how much medical information they want to know, and to have the final say in decisions about their treatments (Windsor et al. 2008). For example, in Opinion 2.1.1 of the AMA Code of Medical Ethics (2001), it is stated, “Patients have the right to receive information and ask questions about recommended treatments so that they can make well-considered decisions about care.” Similarly, in New Zealand, Right 6 of the Code of Health and Disability Services Consumers’ Rights (1996) stipulates that “every consumer has the right to be fully informed”.

However, in many Asian countries including China, a practice described as ‘family-oriented informed consent’ (FOIC) is more prevalent (Ho 2008; Akabayashi et al. 1999; Fan 2015). Families would be informed and make decisions without appropriate engagement of patients in the informed consent process. It is reported that FOIC often happens when the patient is thought to be vulnerable because they are older, female, and/or have a life-threatening illness. FOIC normally happens when the family and the attending doctor suspect that disclosure would cause psychological harm to the patients. To our best knowledge, there is no unified definition of FOIC in medicine and bioethics. In the literature, this approach is variously referred to as family consent, family informed consent, and family-facilitated informed consent (Wang and Nolan 2016; Tamura 2006; Zhang 2012).

Nonetheless, scholars have observed several features of FOIC. For example, patient’s medical information is usually provided first to their family, who then decide whether or not to pass this on to patients. This could be information about the patient’s diagnosis, prognosis, treatment, or the purpose of particular procedures (such as a CT scan or MRI) (Li and Wen 2010; Wuensch et al. 2013). If important decisions need to be made (such as performing surgery or administering chemotherapy), the doctor will approach the family first for a decision. In making this decision, the family may or may not consult with the patient, according to their discretion as to what is best for the patient (Fan and Li 2004; Man 2013). Typically, there is usually one, or sometimes two to three family members who represent the whole family and consult with the attending doctor. These family representatives will then relay the doctor’s treatment or care suggestions to the other family members and the patient (if the family decide to include the patient in the process) (Li and Wen 2010). In this paper, we define FOIC as families having the authority in informed consent, deciding what information should be provided to competent patients and making final decisions.

Studies show that in clinical practice in China, 49%− 70% of informed consent documents are signed by families, with only 23%− 30% signed by patients, and 8.4% co-signed by families and patients (Lian and Lin 2008; Han et al. 2007; Su et al. 2008). A 2018 study reports that 77 out of 124 cancer patients (62.10%) did not know about their diagnosis before chemotherapy (Liu et al. 2018). Another study reports that less than half of the patients (39.4%) are informed of their diagnosis at the time the initial diagnosis is made, while the majority are informed at a much later stage (Wu et al. 2020). Similar practices are also reported in other countries, such as Singapore, South Korea, and Iran (Alden et al. 2018; Radha Krishna et al. 2014; Chong et al. 2015). Some recent studies indicate an increase in patient involvement in decision-making among certain patient groups in some of these countries, though contrary findings have also been reported (Yang et al. 2022, Gu et al. 2023, Sun et al. 2023). An earlier study by two of our team has critically reviewed the apparent cultural differences and unrecognised transcultural similarities in medical truth-telling from a global perspective, particularly a Chinese-Western comparative angle (Nie and Walker 2015).

There are two main justifications for FOIC in China. The first is grounded on purported differences between Chinese culture and Western culture. Proponents of this argument hold that Chinese culture is ‘familistic’, whereas the latter is ‘individualistic’ (Fan 2002). In family-oriented decision making, the decision is made by the whole family collectively and based on what is thought to be best for the person who is unwell. This is an example of Confucius’ filial piety or benevolence, which emphasizes collective autonomy and the obligation to take care of other family members (Tai and Lin 2001). Since family-oriented informed consent is the product of familistic culture, this approach is the most appropriate to the Chinese context and therefore is ethically acceptable in Chinese clinical practice. The second main justification is grounded on the alleged benefits of FOIC (Lin et al. 2017; Liu et al. 2018; Su et al. 2020). The key claim is that bad news, such as information about a terminal diagnosis, could cause anxiety and stress, and so be psychologically and physically harmful (Bagheri et al. 2018). Families worry patients may forego or withdraw beneficial treatments because they do not want to a financial burden on their family (Li et al. 2021). They also worry that patients may develop suicidal thoughts or feelings and end their lives (Zhang 2020; Chen et al. 2017). For such families, maintaining hope is regarded as an important part of achieving better therapeutic outcomes, and being ignorant of the illness is a key aspect of this (Li and Chou 1997). This position is in sharp contrast to the view now held in most Western countries, where individual informed consent is generally regarded as beneficial to patients, by improving the quality of medical decisions, strengthening the trust between doctors and patients, and improving self-management (Bagheri et al. 2018; Qian and Hou 2016; Mondal 2021). In this paper we will focus on this second main justification.

There are a number of studies exploring Chinese patients’ and families’ experiences and perceptions of *informed consent*. However, only a small number investigate and examine the psychological, physical, and emotional effects of FOIC on Chinese patients, and they are mostly quantitative studies (Zhang 2012; Wu et al. 2020; Su et al. 2020). These studies did not find FOIC to be more beneficial to patients. Rather, they indicate that non-disclosure can lead to shorter survival time and does not reduce patients’ levels of anxiety or depression (Su et al. 2020; Qian and Hou 2016). In contrast, patients who are truthfully informed have higher scores for global health-related quality of life, more personal control, and have better “emotional functioning” (Jie et al. 2016). In this study, we investigate and assess the experienced and perceived effects of FOIC for Chinese patients, family members, and healthcare professionals. We build on those recent quantitative studies that have investigated negative experiences associated with this model by using a qualitative method. Through this method, we aim to delve deeper into the harms of FOIC, and to gain a more nuanced understanding of the underlying issues and participant perspectives.

## Methods

This study is part of a wider project exploring how and why families are involved in informed consent in China and critically assessing the two main arguments for FOIC. Semi-structured and in-depth interviews were conducted with 13 patients, 14 family caregivers, and 9 healthcare professionals in Tianjin and Beijing, two megacities in northern China, from August 2018 to December 2018. A total number of eight hospitals were selected. We chose these eight hospitals as they are among the best in China and receive a high number of patient visits every day. In these hospitals, it is much easier to recruit patients and families from different regions, cultural backgrounds, and economic circumstances. The information sheet and the consent form were translated into Chinese, provided and explained to participants. All the participants gave their oral informed consent prior to the interviews. A full list of participants and their demographic information is in Table 1.

**Table 1:** Demographics of participants (*N* = 36)

a. Patient participants					
No	Age	Gender	Qualification	Category of illness	Category of treatment
1	30	Female	Master’s degree	Critical	Surgical intervention/Monitoring
2	58	Male	Middle school degree	Critical	Emergency intervention/Surgical procedure
3	35	Female	Bachelor’s degree	Critical	Chemotherapy/Stem cell treatment
4	30	Female	College degree	Critical	Surgical intervention
5	74	Female	Middle school noncompletion	Critical	Surgical intervention/Ongoing treatment
6	50	Female	High school degree	Critical	Chemotherapy/Stem cell treatment
7	50	Female	Bachelor’s degree	Critical	Chemotherapy/Stem cell treatment
8	50	Female	Master’s degree	Critical	Chemotherapy/Surgical procedure
9	53	Female	Primary school degree	Critical	Surgical intervention
10	50	Female	College degree	Critical	Surgical intervention/Follow-up care
11	35	Female	College degree	Critical	Organ transplant
12	49	Female	Bachelor’s degree	Critical	Surgical intervention/Monitoring
13	92	Male	Unknown	Terminal	Palliative care/Rehabilitation
b. Family participants					
No	Gender	Age	Qualification	Category of illness (of the patient)	Category of treatment (of the patient)
1	Female	Around 80	Middle School degree	Terminal	Surgical intervention
2	Female	Around 60	Unknown	Critical	Device insertion/Surgical intervention
3	Male	49	Bachelor’s degree	Terminal	Surgical intervention/Ongoing care
4	Female	46	Bachelor’s degree	Terminal	Surgical intervention/Ongoing care
5	Male	Around 50	Unknown	Critical	Chemotherapy/Stem cell transplantation
6	Male	26	Undergraduate student	Critical	Chemotherapy/Stem cell transplantation
7	Male	50	Unknown	Critical	Chemotherapy/Stem cell transplantation
8	Female	Around 50	Bachelor’s degree	Chronic/Terminal	Medication/Rehabilitation treatment
9	Male	Around 45	Master’s degree	Terminal	Surgical intervention/Ongoing care
10	Female	29	College degree	Terminal	Surgical intervention
11	Male	Around 60	High school degree	Terminal	Surgical intervention
12	Female	Around 50	Master’s degree	Critical	Medication/Rehabilitation treatment
13	Male	Around 40	Bachelor’s degree	Critical	Palliative care/Symptom management
14	Female	Around 35	Technical secondary school degree	Critical/Chronic	Organ transplant (Kidney)
c. Healthcare professional participants					
No	Gender	Age	Occupation		
1	Male	25	Resident physician		
2	Male	25	Resident physician		
3	Male	40	Associate chief physician		
4	Male	25	Resident physician		
5	Female	30	Nurse		
6	Male	40	Former anesthetist		
7	Male	27	Anesthetist		
8	Female	40	Attending Doctor		
9	Female	40	Attending Doctor		

Patient participants were either consumers of health services at the time of the interviews or had been within 4 years prior to the interviews. They must have been diagnosed with critical or terminal illness and have either experienced or heard others’ experiences of the FOIC approach. We initially aimed to only recruit patients who have experience with FOIC. However, this meant we would have to avoid disclosing medical information during the interviews (e.g. information about the patient’s diagnosis), and hence we aimed to recruit patients who had not been informed initially but later learned about their illness, though still prior to recruitment. The eligible family caregiver participants were those who had taken care of a family member who was diagnosed with terminal illnesses or critical illnesses. Eligible healthcare professionals were those who have either used FOIC in their practice or who have knowledge of colleagues who use it and their reasons why.

The thirteen patients were aged between 30 to 92. Eleven were female and two were male. Seven were between 49–60 years old at the time of the interviews. Three patients had not been truthfully informed initially but were given the correct diagnostic information later by their doctors. These three patients were all female and aged between 50 and 74. The critical or terminal illnesses diagnosed in patient participants include cancer, renal failure, myocardial infarction, and others. Five patients had bachelor’s or higher degrees. The 14 family caregivers were aged between 26 to 80. Seven were females, and seven were males. The nine recruited healthcare professionals included four specialists, one nurse, three resident physicians, and one former anesthetist (who had practice within 5 years prior to the recruitment).

In the interviews, participants were asked how they experience and perceive informed consent in China and the role of families in the practice. All the participants were over 18 years old and competent to consent to participate in the study. Patient and family participants from the same family were preferred, though this was not always possible. Recruitment continued until reaching the criteria of data saturation. Ten healthcare professionals were initially recruited, with one withdrawing for unknown reasons.

Jing-Ru Li conducted the interviews. The interviews lasted between 30 and 150 min and were conducted either face-to-face or via audio calls using WeChat, the most widely used instant messaging application in China. Notes were taken for all of the interviews, and all but two were digitally recorded with the permission of the participants. Jing-Ru Li transcribed all the recorded interviews. Quotes from the interviews that were considered especially relevant to the study were translated into English by Jing-Ru Li and subsequently reviewed by Simon Walker and Jing-Bao Nie.

The findings were analysed using thematic analysis. The coding was done by Jing-Ru Li using NVivo 12. The themes were later checked by Simon Walker and Jing-Bao Nie, and adjustments were made as necessary.

The study was approved by the University of Otago Human Ethics Committee (reference number H18/098).

## Findings

Twenty-five themes in total were identified through the data analysis. In this paper, we report on 22 themes (Table 2) relating specifically to the participants’ views and experiences of FOIC. These are reported under four sections, corresponding to four overarching dimensions: (1) the practice of FOIC; (2) the common reason for FOIC; (3) participants’ negative experiences with FOIC, and (4) participants’ attitudes toward FOIC. In reporting the quotes, we use “P” to refer to “patient participant”, “F” to “family caregiver participant”, and “H” to “healthcare professional participant”.

**Table 2:** Full list of the themes

1. A case-by-case approach
2. Contrasting attitudes toward truth-telling between lay participants and medical professionals
3. Psychological resilience
4. Perceived therapeutic benefits of family-oriented informed consent
5. Preference for being informed
6. The importance of personal choice
7. Factors impacting family involvement in Informed Consent
8. Dissatisfaction and powerlessness
9. The common reasons families choose non-disclosure or partial disclosure
10. The types and content of information usually withheld
11. The types of patients usually not informed
12. The methods doctors use to keep the truth from patients
13. The ways doctors deal with a family’s requirement of non-disclosure or partial disclosure
14. The ways doctors deal with the patient’s wish to be truthfully informed
15. Many patients find out the truth by themselves
16. Patients’ emotional reactions to bad news
17. Staged-disclosure is a common practice in China
18. The ways family caregivers become involved in informed consent
19. The family members usually involved in the informed consent process
20. Cancer is a taboo topic between the patient and family
21. The ways doctors deal with disagreement over treatment decisions between the patient and the family
22. A healthcare proxy is a requirement for patients in many hospitals

### The Practice of FOIC

Our study has found that Chinese families often endeavor to hide bad news from patients. When disclosure was inevitable, families would choose to reveal a small amount of information to the patient over the course of treatment. There were two situations that often prompted families to disclose more: when the patient’s perception of the severity of the illness was close to the truth, and when disclosure would improve the patient’s compliance with the treatment. Therapeutic benefits and psychological distress were the two key factors governing families’ decisions about whether and how much to inform the patient.

Most healthcare professionals in this study would inform the family first and withhold the information from the patient if they were unsure if the family wanted the patient to be informed. One doctor participant said that he would ask the patient to sit in a place where they would not hear his conversation with the family. These participants did report that some Chinese doctors had recently started advising the family to fully inform the patient but added that if the family insisted upon non-disclosure, doctors would normally abide by the family’s request. There were certain situations where doctors might resist such requests, for example, when the patient did not comply with the treatment because of non-disclosure, and if this non-compliance endangered the patient. H06 explained this as follows:

As doctors, we will not encourage the family to inform the patient. Unless non-disclosure affects or hinders the treatment process, we will suggest to the family that they need to truthfully inform the patient. [Otherwise], I do not particularly care about how much information has been disclosed to the patient. (H06)

These practices were confirmed by patient participants. They reported that their doctors normally did not respond to their requests for information if the family had requested non-disclosure beforehand. Often the doctor would refer the patient to their family or find a way to change the topic. If the patient insisted, the doctor probably would disclose some insignificant information, but the information that was suspected to be psychologically harmful would still be withheld. When a dispute over a treatment decision occurred between the patient and the family, most doctors let the patient and the family talk through their disputes by themselves.

A few patient participants said they did not want to be stressed by information about their condition and would prefer not to be told unless the information was essential for better health outcomes. P08 described this unwillingness as follows:

I didn’t feel like talking about my illness [when I was sick]. The pain got worse and worse as the treatment went on. The operation was okay, but the side effects of chemotherapy… When your body was not well, your mood went bad too. When you were unwell, [the conversations about the illness] were essentially about death. (P08)

Families certainly did not initiate conversations about the patient’s condition. Sometimes, even if the patient wanted to talk about their illness, the family would be reluctant to engage with the topic. P01 explained the reluctance in this way:

I don’t know how to describe that feeling. When we had to discuss the treatment options, we would talk about these things. But as soon as the operation was done, we talked no more about it. It feels like, if you don’t talk about it, it never happened. (P01)

P09 made a similar remark:

Even if someone knew about my illness, they never talked about it with me, not even my family. It is okay if I bring it up, but other people [should not bring it up]. Many people had this feeling. (P09)

Any information that might lead to psychological distress would usually be withheld, such as a diagnosis of malignancy, serious risks of the treatments or anaesthesia, and bad prognosis. Benign diagnostic results would be revealed as they were psychologically harmless, and so were the benefits of treatments and a positive prognosis. F04 explained that:

In terms of negative information, if it was not of great relevance to decision making, I would do my best not to talk about it. I felt it was meaningless to tell her about the 3-year mortality of her illness. That information had nothing to do with her recovery, only causing psychological trauma. (F04)

Several medical professional participants stated that patients who were woman, older people, and people who are less educated would have more difficulty understanding medical language and were more likely to be overly worried about their health. The three patient participants in this study who were initially not informed were all female. One (P05) is an older person. One (P09) has only primary school qualification, which is considered “less educated”.

### The Common Reason for FOIC

The majority of participants across the three groups reported ‘therapeutic benefits’ as the main reason for FOIC, either in their own cases or the cases they had heard about. They believed non-disclosure of information about a life threatening or terminal diagnosis would reduce or avoid psychological harm to the patients and preserve hope and thereby increase the chances of a fast and full recovery, and vice versa. A recurring theme in their discussion of these benefits is “psychological resilience” (the original wording in Chinese is 心理承受能力, *xinli chengshou nengli,* literally meaning the mental capacity to bear bad news). Participants generally believed that patients who are less psychologically resilient would experience more distress from the information and benefit less from the treatments. One family participant admitted that as non-disclosure decisions were not discussed with patients it was hard to know “if the patient wants to know the truth or not” (F10). However, given this uncertainty, her preference was not to inform the patient, as she regarded this as the safer option.

Participants described three specific risks of less psychologically resilient patients being truthfully informed: that they would reject beneficial treatments, be less compliant with treatments, and consider ending their lives. For example:

I feel like this [non-disclosure] is a good thing. When you tell the patient he has cancer, immediately, he can't eat or sleep. If the patient's digestive ability becomes poor, his nutrition cannot keep up, the patient will not pull through. (F14)

Sometimes patients can have a weak mind or do not want to be a burden on the family and thus reject the treatment. If that is the case, I think it should be the family [who makes the decision]. (P06)

Several patients and family caregivers claimed that they had heard of or witnessed cases where distressing truths hastened the patient’s death. P02 shared the following story where he believed the patient was severely distressed by the bad diagnosis and stopped eating. The patient miraculously restored appetite after he learned the diagnosis was incorrect, which was mentioned by participants to prove truthful disclosure is harmful by contrast.

My Qishu (the seventh uncle in the family) was suspected to have stomach cancer. He travelled from Baotou [a city in Inner Mongolia] to Tianjin [for further examination]. He was not even able to walk. He was bald and had big eyes. He looked like he was about to die. The doctor [at Tianjin General Hospital] checked on him and said it was merely gastritis to my mother. My mother then told Qishu it was not cancer, it was gastritis. My Qishu immediately said to my mother: “I am hungry.” [By then], he had not been eating for days. When all the treatment was done, he was glowing with health. [However], if he continued the treatment in Baotou for another two weeks, he would have passed away. (P02)

F01’s story had the opposite outcome. The patient reportedly died within a month of learning about his terminal diagnosis.

My grandpa was 76 years old. He was a countryman. He was hard-working and diligent. He was suddenly having a bad appetite and came to Tianjin to see a doctor. He was hospitalised in Tianjin Renmin Hospital for a couple of days and then discharged. As soon as he was home, he could not get out of bed. It was precisely a month from his coming to Tianjin for consultation to his death. I was little then. I did not know what illness he had. I only knew he was not eating and not drinking [after being truthfully informed]. (F01)

### Participants’ Negative Experiences with FOIC

Some participants reported negative healthcare experiences as a result of FOIC. A patient participant believed “her treatment got delayed” due to the concealment of the true diagnosis. She believed she “would have recovered much faster” if she had been fully informed earlier in the treatment course. P06 was diagnosed with acute myelogenous leukemia (AML) M5b. Her husband told her that she had “some problems” but would recover fully with some infusion treatments. When the doctor recommended a bone marrow transplant in the first course of treatment, P06 rejected it because she believed what her husband had told her, and so thought a bone marrow transplant was unnecessary. With each subsequent course of treatment, the doctor repeated the recommendation of a bone marrow transplant, and each time P06 rejected it. It was not until the end of the fifth course of treatment that P06 got suspicious and – with some investigation – discovered the seriousness of her condition. By then, it was already more than a year since she was first diagnosed. Knowing the truth about her diagnosis, P06 finally accepted the bone marrow transplant.

Several patient participants spoke of feeling profoundly hurt by their doctors’ or family’s failure to inform them. The language they used conveyed a sense of abandonment or betrayal. For example, some patient participants spoke about feeling abandoned when their family or doctor ignored their requests for accurate information, or when they refused to respond to their attempts to discuss their illness or feelings. P09 described how angry and upset she was when she learned she had not been informed:

I yelled at my husband when I just found out [the truth]. I blamed him for lying to me. When I heard this word [referring to cancer], I was quite emotional. It’s not possible to conceal the truth completely. You cannot *meng zai gu li tou* [keep the patient in darkness]. There were many things that they [referring to her husband and daughter] did not tell me. After the surgery, I felt my leg was so numb. I felt this part of the flesh stiff. I wondered what happened to my leg. They told me it was because I was leaning on that leg for too long. They told me to massage the leg. Later I knew that was not the reason. Until now, I still feel that blood does not flow smoothly around this area. (P09)

Several patients and family caregivers spoke of how they felt abandoned by doctors when they adopted a social norm of ‘keeping the family affairs within the family’ and left them alone to make decisions. They had to manage the uncertainties and complexities of the decisions by themselves.

I told my doctor, ‘You don’t need to look for my husband all the time. His psychological resilience is no better than mine’. I said, ‘You could tell me directly, I can cooperate with the treatment.’ When I said this, the doctors and nurses were just smiling. (P06)

The sense of abandonment could also occur when they were not given enough time to decide. For example, P04 remarked:

There were moments I had many questions for the doctor. However, I had too many emotions. When you go through this situation, you will think about a lot of things and feel anxious, doubtful, and frightened. You cannot even stay rational and think about your right to this information. Maybe for one thousandth of a second you think of [the right to information], but the next second your mind will be filled up with other emotions. This is not something that you can decide. (P04)

Several patients felt they were forced to sign the informed consent document because it was necessary to initiate treatment.^1^ P09 observed:

If you want to have [surgery], you need to sign [the informed consent document]. Otherwise, they will not perform the surgery. Most hospitals have this regulation. (P09)

Several healthcare professionals also spoke of being dissatisfied with FOIC. They described feeling ‘stressed’ and ‘upset’ when they had to conceal information from patients. They would also ‘worry’ about accidentally slipping some information to patients and thereby upsetting families. H01 said:

Non-disclosure causes a lot of stress to doctors. As resident physicians, we need to do department rotations. Sometimes the resident physician is new to the department and doesn’t know that they should keep the truth from one patient. If the doctor who is on duty fails to warn the new resident physician about this, and the patient approaches the doctor to ask for the diagnosis, then… If you let slip the truth, how will the family treat you? (H01)

To some of them, practicing FOIC was ‘a tough task’. They were also ‘disappointed’ that the healthcare proxy scheme, which was supposed to protect patients’ interests had turned into a formality in practice. H04 remarked:

This is merely a piece of paper in reality. You can view it as a formality… even if your husband signs the informed consent document, when a decision needs to be made in the middle of your operation, it must be your parents along with your relatives who make the decision together. (H04)

Several healthcare professionals reported that patients could be left untreated or have delayed treatment due to family disputes. One of them mentioned a story they read from news in which the young female patient was abandoned by both her maiden family and her husband’s family because the medical expenses were too high to pay. The patient’s health care was jeopardised by this family tangle.

### Participants’ Attitudes Toward FOIC

The majority of patients and family participants believed that FOIC was generally appropriate, because of the assumed benefits of non-disclosure. Only a few patients and family caregivers firmly believed patients should have the final say. However, when questioned further about their reasons for favoring FOIC, those participants agreed that contextual information was also important, and that a case-by-case approach would be more appropriate. For example, F12 said:

I think whoever [either the patient or the family] knows the patient’s condition best has the final say. When the patient’s life can be saved if the patient receives the treatment recommended by the doctor, but the patient declines [the treatment], the patient’s decision should not be respected. (F12)

Similarly, F03 explained:

I think it depends on the people in question. My mother has a religion [referring to Buddhism], and she has already been informed of her illness before the operation. So, it is not necessary to withhold the truth anymore. However, if the person is very vulnerable, once you inform them, they will panic. For this kind of patient, it is better to withhold the information. (F03)

In contrast to the patient and family participants’ somewhat positive attitude toward FOIC, most of the healthcare professional participants believed that *patients* should have the final say in informed consent. When asked whether it was true that bad news hastens death, prompts suicide, or causes a patient to reject beneficial treatments, they said this happens rarely. Instead, they regarded this as an important part of clinical practice. One important reason for this was the “therapeutic benefits” of informed consent, including better self-management, improved compliance with the treatments, and better-informed decisions. H01 explained:

The patient [who is truthfully informed] can communicate with other fellow patients. Because if there is one patient who does not comply with the treatment, like refusing to eat gao ya fan [meals cooked with a pressure cooker] because of its bad taste, [and they get infected], this kind of lesson is powerful. Another example is that, because we cannot provide detailed information [to patients and their family] in the publicity and education programs, like how to purchase medications provided in our hospital and how to do daily care, they can communicate with each other or search for the information on our official WeChat Account. For example, they can communicate with each other to discuss how to take a hip bath after the operation. Because they are informed, they become more proactive. They will understand a lot of medical terms. Sometimes they cannot fully rely on their family to take care of them, as their family cannot do everything. Patients have to manage by themselves. (H01)

H05 said:

Cancer treatment is special. The condition may become even worse. The patient will feel worse after getting the treatment. Therefore, the extent to which the patient understands the prognosis is very important. If you inform the patient, although the truth will shock them at that moment, they will cooperate more fully with the following treatment. [Besides], for those who are really rational, if they have a good life plan, they can have fewer regrets in the last stage of their life. (H05)

Another purported benefit of FOIC is that it protects female patients, who are considered to be more psychologically vulnerable in dealing with the stress of bad news than male patients. On this point, a few healthcare participants stated that male patients could equally become upset by bad news. They stated that female patients are willing to show their emotions and talk more about their feelings, whereas male patients tend to hide their feelings in order not to show weakness. However, this should not be taken to mean that males are stronger than females in dealing with bad news.

A few healthcare professional participants admitted they were conflicted about the harms and benefits of FOIC. For example, H08 echoed the concerns described by patients and family members:

I feel like, for this patient, if he had not been informed at the beginning, what would have happened to him? I think to myself that, if I had not informed him, would his illness have progressed slower? I also think to myself that, if I tell patients the truth, and their lifespan gets shorter, there will be many things left undone... I am conflicted about this. (H08)

Nonetheless, when participants were asked if they would choose FOIC if they were the patient, the majority in all three participant groups said “No”. This apparent inconsistency was explained by the notion of psychological resilience. That is, the majority of the participants believed they personally would be “strong enough to handle the bad news”, and that patients have the right to be informed. F05 said:

Of course, I would want to know. Everyone has different psychological resilience. I would [prefer] to make my own decisions. Don't let others decide your fate. (F05)

P10 said:

We are the masters of ourselves. Only you know yourself best. My husband is merely the person whom I share a roof with. We take care of each other, but we don’t decide for each other. The only things that can keep us alive in our life journey are our soul and our body. We should not rely on other people. It is not right to depend on other people to make decisions for you. (P10)

The few participants who preferred FOIC for themselves later revealed that they would accept being informed as long as it was done in the ways *they preferred*. For example, P11 said she preferred a gradual method:

I hope to only know a little bit. I hope they do not inform me about serious things. I hope to get informed slowly, very slowly. (P11)

P11 also indicated she was not rejecting the idea of disclosure as a whole, rather, it was about having the right person informing her. She described getting informed by doctors of terminal diagnosis were “like judges announcing the death penalty to the accused”. She would prefer to be informed by her family.

The findings also indicated that patients’ reactions to bad news were not always negative as many presupposed. They were various and complicated instead. While they were often initially negative, in most cases the patient would later feel positive about being informed. Among the 13 patient participants, twelve described feeling negative emotions at the time they were given bad news. They reported being frightened, shocked, stressed, and angry. Three said they initially refused to accept the truth. However, eight patients reported a subsequent change of mindset, whereby they accepted their situation and need for treatment. During the interviews, it was observed that patients who were truthfully informed were more likely to receive help from other patients. Some patient participants stated that they would talk about their concerns with fellow patients and encourage each other to overcome the physical pain and the psychological stress.

Participants spoke about the relief they felt when the treatment worked:

[Initially], I was turned upside down. I felt wronged. I had strong reluctance. I had many doubts. I had grievance. I was angry. It was complicated. Gradually, as I was close to the end of the treatment, I felt like I could see the dawn in my life. I felt good. I made it through. (P08)

P05 was the only patient who reported herself to have reacted positively when she first received bad news. She said she was not frightened as she had reached an age where she did not fear death anymore. However, interestingly, when the interviewer cross-checked with her daughter, the daughter said her mother did not actually take the bad news as well as she reported. Both she and her brother (P05’s son) felt she had been upset at the beginning.

## Discussion

### The Under-Recognised Harms of FOIC

Although it is commonly believed that the intervention of families in informed consent will increase benefits and reduce harm to patients, the experiences of many participants in the present study, along with other existing studies, reveal that this is often not the case. Family intervention, even though driven by good intentions, could be harmful to the patients in various ways, and some unintended harms could occur. A previous socio-ethical work has pointed out some real and potential harms involved in the lack of adequate informed consent and the practice of non-disclosure and indirect disclosure through family members in the Chinese context (Nie 2013, Chapters 5 and 7). The present study has identified and underscored the following three main harms and risks associated with the family-oriented model of informed consent.

One of the harms of FOIC identified by participants is the potential for a delay in treatment. A delay in treatment is often detrimental to patients, and in some cases can be life-threatening. This can happen when the patient is not provided with information that would be important to their decisions. This was demonstrated in patient P06’s case. It can also occur when the family disagrees with the doctor over treatment plans and rejects the treatment that would be beneficial to the patient, as mentioned by several healthcare professional participants. Along with a number of examples of this in prominent news stories,^2^ studies show that family hesitation in making decisions is one of the most significant reasons causing delays in emergency treatments in China (Liu et al. 2020; Chen 2018; Tan et al. 2017). Delays in treatment as a result of FOIC have also been reported in other countries. For example, in the Kingdom of Saudi Arabia, family-induced non-disclosure leads to a delay in cancer treatment and difficulties in cancer care management (Wazqar et al. 2017). A similar finding was reported in Morocco (Soliman et al. 2019). Participants of the study said they were hesitant about getting treated because of their financial dependence on their family. The key problem is that under FOIC the patient’s interests can be effectively ignored. As Hyun has pointed out, if the patient is at the lowest level of a decisional hierarchy, there is the possibility that the patient’s interests will be neglected in favor of the overall interests of the family or the personal interests of the family decision-maker (Hyun 2002).

The second significant harm is the personal hurt experienced by patients, often described as a sense of abandonment. Patient participants described how they felt abandoned because of the conspiracy of silence adopted by families and doctors as a team. Vliet has argued that the confusion and stress caused by the uncertainties about the illness are worse than the distress of hearing bad news (van Vliet et al. 2013). When families and doctors avoid talking about the patient’s illness, patients are left alone to manage the anxiety generated by their unanswered questions. This anxiety is often compounded when they inadvertently discover some information on their own and are unsure of what it means. Katz (2002, 208–209) highlighted this kind of abandonment of patients in his book *The Silent World of Doctor and Patient*. He discussed how patients often feel abandoned when doctors treat them as children, withholding crucial information from them and attempting to soothe them with a “pat on the back” and by saying “Everything will be all right” (Katz 2002, 210). The findings of this study indicate how this sense of abandonment is not only related to doctors and patients, but also concerns patients and families, and can be generated by FOIC. Katz noted that the feeling of abandonment could be more painful than physical abandonment because it was more difficult to identify, and hence patients do not know how to manage their emotions. He observed that they may start to blame themselves for being greedy and needy (Katz 2002, 210). This would explain why none of the patient participants explicitly described themselves as having been abandoned. Even after they forcefully expressed their negative feelings in the interviews, they often reverted to a position of equanimity and tried to smooth over the problem. They seemed to lack an understanding of why they felt so strongly.

The third significant harm is poorer patient self-management. Self-management is a patient’s “ability to manage the symptoms, treatment, physical and psychosocial consequences and lifestyle changes inherent in living with a chronic condition” (Barlow et al. 2002). Lack of information regarding diagnosis, prognosis, treatment, and care plans can impair a patient’s ability to manage their condition. By contrast, involvement in informed consent would encourage patients to manage these tasks themselves, and patients will transform from passive recipients to active participants. As reported earlier, several patient and healthcare professional participants mentioned patient involvement in decision making as a good way to improve self-management. Studies have found that active participation can improve patients’ knowledge about cancer, reduce psychological stress, and increase health-related quality of life (Barlow et al. 2002; Kim et al. 2017; Huang et al. 2020; Cuthbert et al. 2019).

As revealed in the findings, patients could easily obtain information regarding self-management through communication between patient peers. Those patients who have more experience and knowledge can share their wisdom with those who have less. Patient communities improve self-efficacy, change health behaviors, and empower patients to increase their knowledge (Willis and Royne 2017; Willis 2016). The cultural taboo against speaking about death and cancer in China, revealed in the interviews and other literature, would normally stop patients from talking about their situation with their family. However, patient communities can provide important emotional support. Patients may find it easier to open up to their patient peers than their family because they have had similar experiences (Wang et al. 2015; Zhou 2018). It has also been found that the connections made with other cancer patients can relieve the sense of loneliness and create a sense of belonging (Yu and Wang 2020). Patients who are not informed of their condition and excluded from decision making are unlikely to access these benefits.

### The Purported Benefits and Harms of FOIC

The findings suggest that the purported benefits of FOIC could, at least in some cases, be false, and that many of the apparent and assumed harms of informing patients are often overstated.

Participants across the three groups asserted that the psychological resilience of Chinese patients was generally low. However, this does not fit with the findings regarding patients’ experiences. Although most patients initially reacted negatively to the bad news they received, they were able to recover from this and would then take the doctor’s healthcare advice and accept recommended treatments. This included the patients who are often presumed to be particularly vulnerable. None of the medical professionals in this study reported cases where disclosure had jeopardised a patient’s health. It is true that some patients regarded their illness as a ‘taboo subject’ and were reluctant to receive information. However, this does not mean that all Chinese patients are too vulnerable to be honestly informed about their health. To begin with, this is not true of the patients who participated in this study. More significantly, there are studies showing that older Chinese people, who are often assumed to be psychologically weaker, are not, or are less likely to be, afraid of death (Ming-lin and Lang 1998; Chen et al. 2017). We are not aware of any studies directly comparing the psychological resilience of Western and Chinese patients, but there is no obvious reason why there should be a difference. Also, our enquiry into the participants’ reasons for preferring non-disclosure indicates that those patients would in fact be open to honest information, so long as it was provided in the manner that they preferred. This suggests that a key consideration in gaining informed consent is not whether or not to provide information but understanding how patients want to be informed.

Many participants reported that they have heard about cases where truth-telling seemed to cause illness or hasten death. However, these anecdotes are likely to be unreliable, and the broad claim is not supported by solid evidence, or the views of the healthcare professionals’ in this study. On this purported link, Katz’s (2002, 220) made the following remark:

Even when disclosures of prognosis have been made, it is not clear whether death was the result of physiological or emotional causes. Instead, it may have been due to physiological causes such as starvation and dehydration. It is most questionable to draw any conclusive inferences about the inadvisability of disclosure of dire prognosis.

On the relation between truth-telling and suicide, while there are studies showing that cancer patients are at higher risk of suicide, there is no solid evidence indicating that being informed is the *direct reason* leading to suicide, or that cancer patients who are truthfully informed are more likely to end their own lives (Du et al. 2020; Spoletini et al. 2011; Anguiano et al. 2012).

On the belief that female patients are more susceptible to psychological harm by truthful disclosure than male patients, there is a little research on the sex difference in psychological resilience in response to a bad diagnosis, and the results available are mixed. A study in Taiwan found that older females are less resilient than older males due to social norms or values (Fergus et al. 2024). A similar result is shown in a study in Pakistan, which reveals that males with burns are more resilient than females (Masood et al. 2016). However, there are other studies showing that females can be more resilient than males, or that females and males have the same level of resilience (Moser and Dracup 1995; Arı and Çarkıt 2020; Behera et al. 2020). More importantly, several studies report that female and male patients equally prefer to be truthfully informed. Studies in Taiwan and India show that both female and male patients would prefer full and frank disclosure (Chen et al. 2018, 2020; Mondal 2021). In Iran and Malaysia, studies reveal no significant association between patients’ attitudes toward truth-telling and their sex (Bagheri et al. 2018; Eng et al. 2012). To our knowledge, there is no such study done in mainland China. However, the cultural norms related to sex in the countries mentioned are similar to those in China, and so the results may be applicable to Chinese patients. Therefore, the scientific evidence available provides little support for the claim that female patients in China are less psychologically resilient than male patients. Moreover, females are traditionally more subject to undue pressure from their family due to the patriarchal culture. This suggests that extra attention should be given to potential coercion when a family requests that a female be excluded from the informed consent process.

As this is a qualitative study, the results cannot be used in isolation to make claims about Chinese patients in general. What the findings provide is insight into the underlying experiences and perspectives of some Chinese people in relation to informed consent, the layers of complexity involved, and of the possible ways that apparent benefits and harms may be understood. China is a highly populated country with a significant internal plurality. Each province, city, and even family may have a distinct culture. Future studies, using quantitative research methods, could further explore the relationship between bad news and psychological resilience among Chinese patients across different regions and ethnic groups. Though we were not able to recruit more non-informed patients in this study, all of the patient participants had experience of FOIC, and were able to provide information about how and why it is practiced in China.

The findings reveal tensions in how patients, family members, and healthcare professionals view informed consent, particularly regarding when and how information should be provided to patients. Some of these tensions could be addressed by training doctors how to communicate bad news and discuss these matters with patients and families. Further research could examine how informed consent is taught in Chinese medical schools, both to assess whether the teaching is consistent with the current law and informed by up-to-date research on patients’ and healthcare professionals’ views, and to identify possible improvements that may support future doctors’ ability to sensitively manage these difficult conversations.

## Conclusion

This paper examines how and why FOIC is practiced in China and focuses on the argument that FOIC is more beneficial to Chinese patients than the individual model practiced in most other countries. The findings confirm that the notion of ‘psychological resilience’ is a key factor in this argument but also reveal that patients in China may oppose FOIC for various reasons, including the right to self-determination and informed consent. In addition, they confirm concerns about the ways that FOIC can be harmful to patients and undermine the claim that it is beneficial. The potential harms of FOIC include serious delays in treatment, psychological abandonment, and undermining patients’ capacity for self-management. The findings – in keeping with other available evidence – provide no support for the claim that Chinese people, or female Chinese patients, are less psychologically resilient than others. Overall, there is very little evidence to support the claim that FOIC is more beneficial to Chinese patients, and considerable evidence indicating that FOIC may cause significant harms, and hence FOIC cannot be ethically justified on this basis. Moreover, these findings significantly challenge the ‘cultural difference’ argument commonly used to support FOIC in China. While an earlier work by one of our team critically examined this argument against informed consent and truth-telling in China from a Chinese-Western comparative perspective (Nie 2013, Chapters 5, 6 and 7), further in-depth discussion of the implications for this common argument, based on the empirical findings and ethical analysis of the present study, will be published in a separate paper.

While the Civil Code of the People's Republic of China took effect in 2021 which stipulates patients’ right to informed consent, qualitative research exploring how informed consent is practiced in Chinese medical settings remains limited. Although this study was conducted prior to the Civil Code, its implications are still relevant and provide a solid foundation for future assessments of the impact of this new legislation, as well as exploring ways to align the law with practice.

## Data Availability

The interview transcripts are not publicly available due to the potential risk of participant identification. A summary of anonymized supporting data can be made available from the corresponding author upon reasonable request.

## Footnotes

1 Usually, the patient and the family would be asked to sign the informed consent document and appoint a healthcare proxy as soon as the patient is hospitalised. In most cases, the healthcare proxy took effect from the moment the patient signed the form, irrespective of the patient’s mental capacity. The doctor would stop consulting the patient and instead consult the healthcare proxy. Since there was not a specific legal provision on the legal duty or restrictions of the authority of a healthcare proxy in China, if the decisions made by the healthcare proxy would intentionally or unintentionally harm the patient, the healthcare proxy would not face any legal consequences. This could create problems, as the healthcare proxy was not necessarily the person who best represented the patient’s welfare, and sometimes they were merely assigned by the family to sign the informed consent document but did not have the authority in making final decisions. Moreover, in surgery departments, if the patient declined the requirement, the patient could be refused to get admitted or from getting the operation (unless it was an emergency procedure). One medical professional explained that healthcare proxy agreement was a mandatory requirement in surgery departments as it would reduce their disputes with family caregivers.

2 For example, in 2007 a pregnant woman did not get the emergency treatment she needed and died as a result. The hospital did not provide the treatment because they could not obtain consent from the husband. In 2017, another woman who was about to give birth jumped over the hospital building because her requirement for a C-section operation was disapproved by her family, and the hospital followed the family’s choice and withheld the C-section from her. There is some disagreement about the details of the second case, but nonetheless it shows how a delay in treatment can be caused by FOIC.
